# Medial-Sided Ligamentous Injuries of the Athlete's Knee: Evaluation and Management

**DOI:** 10.7759/cureus.36360

**Published:** 2023-03-19

**Authors:** Garrett Chapman, Neeraj Vij, Robert LaPrade, Nirav Amin

**Affiliations:** 1 Orthopedic Surgery, Spine & Joint Institute, Redlands, USA; 2 Orthopedic Surgery, University of Arizona College of Medicine, Phoenix, USA; 3 Orthopedic Surgery, Twin Cities Orthopedics, Eagan, USA; 4 Orthopedic Surgery, Restore Orthopedics and Spine Center, Orange, USA

**Keywords:** athlete's knee, ligamentous injury, sports surgery, patient-centered care, surgical reconstruction, posterior oblique ligament, ligamentous instability, medial knee

## Abstract

The superficial medial collateral ligament (sMCL) is the most commonly injured ligamentous structure in the knee. The other medial knee stabilizers include the deep medial collateral ligament, the posterior oblique ligament, and the medial meniscus. Medial collateral ligament injuries frequently occur in young athletes. As a result of the good healing capacity of the sMCL, the majority of acute medial-sided knee injuries can be treated nonoperatively with good outcomes. However, missed concomitant injuries can lead to residual laxity and instability of the knee when treated conservatively. When surgical management is warranted, numerous techniques exist, including repair, augmentation, and reconstruction. Recent anatomic and biomechanical studies defining the attachment sites and functional roles of the individual medial knee structures have led to advancements in diagnosis, treatment, and rehabilitation. These studies have allowed for the development of an anatomic reconstruction technique that restores the native stability and load-sharing relationships among the medial knee structures. The purpose of this narrative review is to summarize the recent updates in the anatomy, biomechanics, evaluation, and treatment of ligamentous injuries on the medial side of the athlete's knee.

## Introduction and background

Injuries to the medial knee structures are common with a reported incidence of 0.24 per 1,000 in the United States per given year [1]. The superficial medial collateral ligament (sMCL) accounts for approximately 40% of ligamentous knee injuries [2,3]. The majority of the medial collateral ligament (MCL) injuries are isolated; however, there is a high incidence of concomitant medial-sided knee injuries with increasing injury severity [2]. These injuries are frequently seen in young, active individuals participating in athletics, and typically result from a contact or non-contact valgus force to the knee [3]. Although many medial ligament tears can be managed nonoperatively, there is a lack of consensus as to when surgical treatment is indicated [1,3]. The purpose of this narrative review is to summarize the recent updates in the anatomy, biomechanics, evaluation, and treatment of ligamentous injuries on the medial side of the athlete's knee.

## Review

Anatomy

An understanding of the structures comprising the medial knee is helpful for accurate diagnosis of injuries and for optimizing outcomes when surgical treatment is necessary. Warren and Marshall described the anatomy as three distinct layers [4]. Layer I is composed of the deep fascia, which wraps the medial knee from the patella to the popliteal fossa posteriorly [4]. Layer II includes the sMCL, the medial patellofemoral ligament, and the ligaments of the posteromedial corner (PMC). It merges with layer I anterior to the sMCL and joins with layer III posteriorly to form the PMC [4]. The gracilis and semitendinosus tendons are located between layers I and II. Layer III consists of the medial joint capsule and the deep MCL (dMCL) [4].

The sMCL has an average length of 100.7 ± 9.5 mm and the dMCL has an average length of 26.2 ± 5.6 mm in the meniscofemoral ligament (MFL) portion and an average length of 9.2 ± 1.8 mm in the meniscotibial portion [5]. Alternatively, the medial knee anatomy has been described by dividing it into thirds from anterior to posterior [5]. The anterior third consists of extensor retinaculum fibers and underlying capsular ligaments [5]. The middle third contains a fascial layer along with the sMCL and dMCL [5]. The posterior third consists of the PMC (posterior oblique ligament (POL), oblique popliteal ligament, semimembranosus attachments, and posterior horn of the medial meniscus) [6].

**Figure 1:**
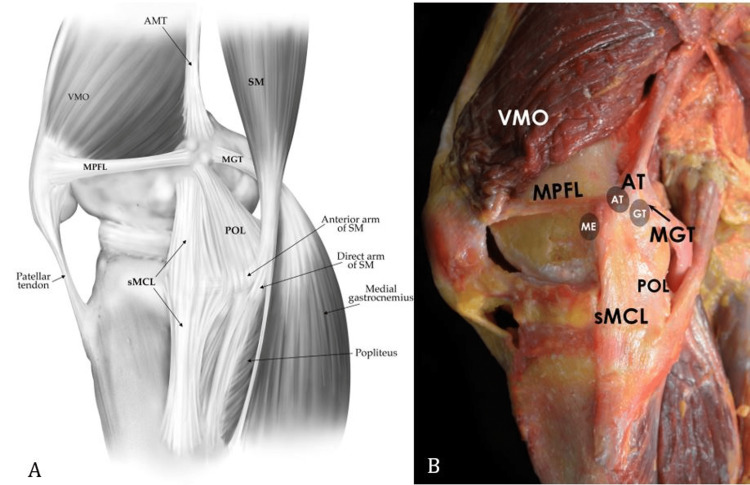
Illustration (A) and photograph of a cadaver specimen (B) showing the medial knee structures. AMT = adductor magnus tendon; AT = adductor tubercle; GT = gastrocnemius tubercle; ME = medial epicondyle; MGT = medial gastrocnemius tendon; MPFL = medial patellofemoral ligament; POL = posterior oblique ligament; SM = semimembranosus; sMCL = superficial medial collateral ligament; VMO = vastus medialis obliquus. Panel A: Adapted with permission from LaPrade RF, Engebretsen AH, Ly TV, Johansen S, Wentorf FA, Engebretsen L. The anatomy of the medial part of the knee. J Bone Joint Surg Am. 2007;89(9):2000-2010 [7]. Panel B courtesy of LaPrade, RF.

More recently, LaPrade et al. quantitatively defined the attachments of the individual medial knee structures (Figure 1) [7].

On the medial aspect of the distal femur, three osseous prominences are consistently present. The medial epicondyle is the most distal and anterior of these bony landmarks [7]. The adductor tubercle is the most proximal and lies at the distal edge of the medial supracondylar line [7]. The gastrocnemius tubercle, the most posterior prominence, is located slightly distal and posterior to the adductor tubercle [1].

The sMCL is the largest and most important structure of the medial knee. Its femoral origin is 3.2 mm proximal and 4.8 mm posterior to the medial epicondyle [7]. There are two locations for tibial attachment. The proximal tibial attachment is 12.2 mm distal to the joint line and is primarily connected to soft tissues over the termination of the anterior arm of the semimembranosus [7]. The distal attachment is 61.2 mm distal to the joint line and attaches directly to the posteromedial crest of the tibia [7]. Between the two tibial attachments, the inferior medial genicular artery and vein run deep into the sMCL.

The dMCL (mid-third medial capsular ligament) is a thickening of the joint capsule deep to the sMCL and consists of two distinct functional units: the meniscofemoral and meniscotibial divisions (coronary ligaments.) The meniscofemoral portion attaches to the femur 12.6 mm distal to the sMCL, whereas the shorter and thicker meniscotibial portion attaches to the tibia 3.2 mm distal to the joint line [7].

The POL consists of three fascial attachments that extend off the distal aspect of the semimembranosus tendon to blend with and reinforce the posteromedial aspect of the joint capsule [7]. It attaches to the femur 1.4 mm distal and 2.9 mm anterior to the gastrocnemius tubercle [1,7]. The three attachments of the POL have been termed the central (tibial), capsular, and superficial arms [7-9]. The central arm, being the largest and thickest, is considered the main component of the POL. It directly attaches to the posterior joint capsule and posteromedial meniscus, reinforces the dMCL, and can be differentiated from the sMCL with its fan-like fibers that course proximally [7]. The POL, the semimembranous tendon and its expansion, the oblique popliteal ligament, the posterior horn of the medial meniscus, and the posteromedial joint capsule comprise the PMC [7].

The adductor magnus tendon serves as an important surgical landmark because it is rarely injured and can be a reference for identifying the femoral attachment sites of the POL and sMCL [7,10]. It attaches to a small depression 3.0 mm posterior and 2.7 mm proximal to the adductor tubercle [7]. Similarly, the femoral attachment site of the medial gastrocnemius tendon is another important surgical landmark. The tendon attaches 2.6 mm proximal and 3.1 mm posterior to the gastrocnemius tubercle and helps to identify both the gastrocnemius tubercle and the attachment of the POL [7,10].

Biomechanics of the medial knee

A thorough understanding of the biomechanics of the medial knee is important to understand the abnormal joint motion that occurs with medial knee injuries. In addition, it aids in the interpretation of clinical exam findings and helps determine the presence of concurrent injuries in the knee. Force measurement and sequential sectioning studies have quantified the function of individual structures and elucidated the intricate load-sharing relationships that exist between structures of the medial knee [11-13]. The key static stabilizers of the medial knee include the sMCL, dMCL, and POL, which provide stability against abnormal valgus motion, internal/external rotation, and anterior/posterior translation of the knee [11]. The key dynamic stabilizers include the pes anserine tendons, the semimembranosus, and the medial head of the gastrocnemius [12].

Normal physiologic valgus is between 0 and 3 mm of medial joint space opening [13]. Normal physiologic rotation ranges from 45° of external rotation and 25° of internal rotation with the flexed knee to 90° and 23° of external rotation and 10° of internal for the knee flexed to 0° [14]. The sMCL serves as the primary valgus stabilizer and consists of two functional divisions, proximal and distal, that have quantitative differences to applied loads. The proximal division serves as the primary stabilizer to valgus motion and a secondary stabilizer to external rotation at 90° of knee flexion and internal rotation at 0°, 30°, and 90° of knee flexion [14]. The distal division serves as the primary stabilizer to both external rotation at 30° of knee flexion and internal rotation at all flexion angles, and as a secondary stabilizer to external rotation at 0°, 20°, and 60° of knee flexion [14]. Biomechanical studies have demonstrated that the two divisions of the sMCL function as conjoined but distinct structures, and surgical repair or reconstruction should restore both components to reproduce the overall function of the sMCL [13].

Previous sequential sectioning studies have shown the dMCL to be an important secondary stabilizer to valgus stress when the sMCL is injured [11]. Similar to the sMCL, the two components of the dMCL each serve various stabilizing functions. The meniscofemoral portion is a primary stabilizer to internal rotation stress at 20°, 60°, and 90° of knee flexion, and provides secondary internal rotation stability at 0° and 30° of flexion [11]. Additionally, the MFL acts as a secondary valgus stabilizer at all knee flexion angles, as well as a secondary external rotation stabilizer at 30° and 90° of flexion [11]. The meniscotibial portion acts as a secondary valgus stabilizer at 60° of knee flexion and provides secondary internal rotation stability at 0°, 30°, and 90° of flexion [13]. Regarding the most common site of failure of the dMCL, several studies are in disaccord. Operative findings in a clinical series by Sims and Jacobson concluded the femoral attachment was the most common site of injury for the dMCL; whereas a cadaveric study by Robinson et al. showed that mid-substance tears were the most common [15]. Despite this lack of consensus, the dMCL has been shown to have the lowest load-at-failure, stiffness, and displacement-at-failure of the three main ligaments of the medial knee [15].

Biomechanically, the POL is a primary restraint to internal rotation at all knee flexion angles with the highest loads experienced at full extension [16]. The POL also plays a secondary role in valgus and external rotation stability, which becomes more pronounced in the setting of MCL deficiency [16]. This was demonstrated in a biomechanical study that reported a significant increase in forces on the POL under valgus stress at early knee flexion with sectioning of the sMCL and dMCL [12].

When an injury to the POL occurs concomitantly with a tear of the sMCL, severe valgus instability can result, which highlights the importance of repairing or reconstructing the POL in severe medial knee injuries [9].

Native healing

Knowledge of the basic science of ligament healing is useful when considering treatment strategies for medial knee injuries. The sMCL has served as the prototypical extra-articular ligament for basic science research due to its ease of identification and abundant blood supply [17]. In response to injury, the sMCL follows the classic healing model that includes hemorrhage, inflammation, repair, and remodeling [17]. Studies analyzing the variables that affect MCL healing have shown that the location of the injury, the severity of the injury (including concomitant ligamentous injury and the resulting instability), and the degree of prolonged immobilization are all important factors [17-19]. Using a rabbit model to examine the effect of injury location on MCL healing, Frank et al. demonstrated injuries near either attachment site healed more slowly than mid-substance injuries [18]. Despite the production of repair tissue that can fill a defect in the ligament, the mature tissue remains mechanically inferior to native ligament tissue [20]. Additionally, ligament healing across larger gaps is structurally inferior to the healing that occurs when the injured ligament ends remain in contact [21].

Numerous basic science and clinical studies have demonstrated the deleterious effects of immobilization on ligament healing and outcomes following injury [17-22]. Biologically, immobilization results in decreased collagen synthesis, a reduction in collagen mass, and increased collagen degradation within the MCL, resulting in part from a transition in the metabolic behavior to a more catabolic state [19]. Conversely, mobilization following injury is beneficial to the healing ligament as evidenced by improvement in the longitudinal alignment of collagen and cells, increases in the tensile strength and ultimate load, and a significant decrease in laxity [17,22]. However, the benefits of active motion on MCL healing appear to be dependent on the overall stability of the joint. Early motion in an unstable knee joint is detrimental to the MCL healing process and ultimately leads to chronic laxity [22].

Diagnosis

History

MCL injuries most commonly occur via a contact injury; however, non-contact injuries have been described particularly in skiers [23]. The mechanism may be a direct blow to the lateral side of the knee while the foot is planted, which occurs in contact sports such as rugby and football [1]. A second injury pattern occurs when a valgus force is combined with the external rotation of the tibia [2]. This pattern often occurs in pivoting sports such as soccer, basketball, and skiing. Patients may report pain and swelling over the medial knee, loss of motion, and pain with weight-bearing, while others can experience significant side-to-side instability [10]. A breakdown of the clinical appearance along a time course is provided in Table 1.

**Table 1:** The clinical appearance of medial collateral ligament injuries based on the duration from the initial injury.

Time course	Clinical appearance
Acute (minutes - 24 hours)	Felt a pop, fell to the ground, immediate pain. Able to walk, unable to run or cut. Painful to flex and extend.
Subacute (1 - 6 days)	Swelling, medial knee pain, knee stiffness. Able to walk, unable to run or cut.
Healing (1 - 8 weeks)	Improved pain. Progressive reduction in swelling. Improving range of motion. Able to walk, unable to run or cut.
Healed (>8 weeks)	Full return of function. May have some residual laxity.

Classification

Several classification systems exist for MCL injuries. The lack of standardized terminology has caused confusion and difficulty in the comparison of outcomes. Most widely used classification systems are based on the amount of medial compartment gapping present with a clinician-applied valgus stress performed with the knee in 20-30° of flexion [1-3]. The grading scale established by the American Medical Association is one of the most widely used [24]. Under this system, a grade I, first-degree tear presents with localized tenderness and no instability [24]. A grade II, second-degree tear presents with localized tenderness and an incomplete tear of the MCL [24]. Pathologic laxity may or may not be present, but a definite end point is present. A grade III, third-degree tear presents with complete disruption of the MCL complex with laxity and no definite end point. Grade III injuries can be subdivided based on the amount of laxity observed during valgus stress with the knee flexed to 30° [24]. Grades 1+, 2+, and 3+ correspond to subjective gapping of the medial joint line of 3-5 mm, 6-10 mm, and >10 mm, respectively. Alternatively, Fetto and Marshall described a classification system that evaluates medial knee laxity at both 0° and 30° of knee flexion [2]. Injuries are divided into grade I (no valgus laxity), grade II (valgus laxity at 30° of knee flexion), and grade III (valgus laxity at 0° and 30° of knee flexion).

Physical Examination

The physical exam remains a valuable tool for the diagnosis of MCL injuries (Table 2).

**Table 2:** The physical examination of medial collateral ligament knee injuries based on duration from the initial injury. sMCL = superficial medial collateral ligament; dMCL = deep medial collateral ligament.

Time course	Physical examination
Acute (minutes - 24 hours)	Swelling: Intra-articular effusion vs. generalized edema
	Tenderness to palpation of the medial knee:
	1) Distal femur (proximal sMCL origin)
	2) Proximal tibial (distal sMCL insertion)
	3) Medial joint line (sMCL mid-substance injury vs. dMCL vs. medial meniscus)
Subacute (1 - 6 days)	Repeat the above in the context of decreased swelling
Healing (1 - 8 weeks)	All of the above and the functional range of motion testing
Healed (>8 weeks)	All of the above with stability and strength testing

This begins with a visual inspection of the skin and soft tissues, looking for the presence and location of swelling, abrasions, ecchymosis, hemarthrosis, or an effusion [1-3]. It is important to distinguish between a medial swelling and a true joint effusion within the suprapatellar pouch [1]. Dimpling of the skin over the medial knee can be a sign of an entrapped MCL or an irreducible knee dislocation, whereas an effusion may indicate a concomitant intra-articular pathology [25]. Careful palpation of the medial knee structures, including along the entire length of the MCL, can yield important information regarding the location of the injury [3]. A thorough assessment of the patient’s neurovascular status is mandatory, especially for multi-ligamentous injuries or knee dislocations, and any vascular abnormality should prompt a vascular surgery evaluation.

Valgus stress testing is the mainstay of the physical examination [1-3]. The test should be performed with the patient positioned supine at the edge of the examination table to allow the knee to flex with the thigh supported to prevent any rotation at the hip [3]. A valgus stress is then applied to the leg with a fulcrum on the lateral knee while the examiner palpates the medial joint line to determine the amount of gapping present. The test is performed with the knee in full extension and 30° of flexion to evaluate both the sMCL and PMC [2]. Valgus laxity at 30° of flexion is suggestive of a tear of the sMCL, while laxity at 0° and 30° of flexion suggests a complete injury of both the sMCL and PMC and should raise concern for concomitant cruciate ligament injury [2]. It is also important to assess the quality of the end-point to differentiate between a partial and complete medial knee injury. Examination of the contralateral leg for side-to-side medial joint line gapping differences is mandatory. It is very important to perform a full knee exam, including a posterior drawer, a varus stress test, and a thorough exam of the medial meniscus. Sims and Jacobson reviewed 93 knee injuries and found additional concomitant injuries in 88%, including an associated anterior cruciate ligament (ACL) tear in 78% [15].

Assessment of anteromedial rotatory instability (AMRI) is also very important. AMRI is tested through the anteromedial drawer test, which is performed by externally rotating the foot 10° to 15° and applying a gentle anterior translation to the knee in both 90° and 30° of flexion [26]. The amount of anteromedial subluxation of the medial tibial plateau is visually assessed. The degree of AMRI can give insight into concomitant POL and/or PMC injuries [15,26].

Imaging Studies

Although frequently normal in an acute injury, all patients should have plain radiographs to evaluate for joint subluxations, joint dislocations, avulsions, osteochondral injuries, and other fractures. Anteroposterior (AP), lateral, and valgus stress radiographs are the most commonly ordered [1-3,27]. A common method of obtaining an adequate AP view of the proximal tibia is to have the knee fully extended, with an X-ray beam angled at 20 degrees [3].

Valgus stress radiographs allow for quantitative grading of medial knee injuries [27]. Stress X-rays are commonly used to follow the healing of both nonoperative treatment and surgical reconstruction, and are a useful adjunct to the physical exam in diagnosing chronic medial knee injuries. These radiographs are performed with the knee in 20° of flexion and a comparison of medial compartment gapping is made to the contralateral side. LaPrade et al. found that a grade III injury to the sMCL increased medial compartment gapping by 1.7 and 3.2 mm at 0° and 20° of knee flexion, respectively [27]. A complete medial knee injury involving the sMCL, dMCL, and POL increased gapping by 6.5 and 9.8 mm at 0° and 20° of flexion, respectively [27]. Instrumented valgus stress radiographs have been described in the literature [28]; however, there does not appear to be any consensus regarding their integration into standard radiographic evaluation.

In a patient with chronic medial-sided knee pain after an MCL injury, X-rays may show a Pellegrini-Stieda lesion, characterized by intra-ligamentous calcification near the femoral origin of the MCL [29]. Although these are commonly incidental findings in patients with a remote history of MCL injury, they may be a source of pain [29]. For chronic injuries, full-length standing radiographs allow for the evaluation of overall limb alignment and joint space narrowing, which have important implications for both treatment and expected outcomes [3,29].

Magnetic resonance imaging (MRI) is now commonly obtained in the evaluation of medial-sided knee injuries, as it allows for the determination of the degree, location, displacement, and other concomitant ligamentous injuries. In one study, the diagnosis of MCL injuries via MRI was found to have an accuracy of 87% [30]. The MCL is best assessed on the coronal images using T1 and T2-weighted sequences [29]. Bone bruises occur in up to 45% of patients with isolated MCL injuries and are predominantly located on the lateral femoral condyle and lateral tibial plateau [31].

Treatment

Despite the medial-sided knee stabilizers being the most injured ligaments, controversy exists throughout the literature regarding the treatment of these injuries [32-35]. Conflicting and overlapping classification schemes, along with a lack of standardization in reported outcomes, have been sources of confusion with the interpretation of treatment results [32]. Most studies include an assessment of valgus stability; however, validated outcomes scores are only reported in more recent studies [33]. An additional source of disparity in reported results relates to the inclusion of both patients with isolated and multi-ligamentous injuries in the same study population [34]. Given the lack of agreement among investigators and the numerous confounding variables regarding medial-sided knee injuries, it is challenging to provide a comprehensive evaluation of treatment strategies.

Nonoperative Management

Due to the healing capability of the sMCL, nonoperative management is the mainstay of treatment for isolated, grade I and II injuries [34]. Treatment focuses on early rehabilitation with a controlled range of motion and progressive strengthening exercises, functional bracing, and protected weight-bearing with the use of crutches [34]. Patients can return to sports when their pain has resolved, no effusion or instability is present on examination, and strength, proprioception, and range of motion are equal to the contralateral extremity [32]. Several rehabilitation protocols have been described, each reporting successful results [32-35]. While many similarities exist among the various protocols, the specifics vary according to the treating provider. To our knowledge, there is no prospective study evaluating the different protocols.

Overall, the results of nonoperative management of isolated grade I and II MCL injuries are consistently favorable [32-35]. In a prospective study of 38 patients with isolated grade I and II MCL injuries treated with early functional rehabilitation and bracing, Lundberg et al. reported 74% had regained nearly normal knee function by three months [35]. At four years, the median Lysholm score was 100 and at 10 years, 92% of patients had good to excellent functional scores. However, the authors noted a decrease in sporting activity levels at the late follow-up evaluation and radiographic evidence of early osteoarthritis in 13% of patients [35]. In high-level athletes, conservative treatment has proven to be effective and allows for a quick return to play. In a study by Derscheid et al., collegiate football players with grade I MCL injuries returned to full, unprotected participation after an average of 10.6 days lost; players with grade II injuries returned after 19.5 days [34]. The conclusion is that there is no demonstrable benefit to operative treatment in these patients.

The management of grade III medial knee injuries has been more controversial, with studies supporting both surgical and nonsurgical treatment [36-39]. For acute, isolated grade III medial knee injuries, initial nonoperative management is generally recommended and associated with favorable results [36-39]. In a prospective study, Indelicato et al. compared the results of primary surgical repair to the nonoperative treatment of isolated grade III MCL tears, which were confirmed with an examination under anesthesia and arthroscopy [37]. The rehabilitation program was the same for both groups, except patients managed nonoperatively were immobilized for two weeks (versus six weeks for the operative group). No difference was seen in the combined subjective and objective scores, suggesting surgical intervention did not offer any advantage, and in fact, rehabilitation progressed more rapidly with a faster recovery of strength with nonoperative treatment [37].

Prolonged immobilization has fallen out of favor as many modern rehabilitation protocols emphasize early motion with an overall focus on controlling edema, restoring knee range of motion, and regaining quadriceps function and strength [32-39]. The early use of a stationary bike is encouraged because it is similar to a continuous passive motion device, which has been shown to stimulate an accelerated healing response with complete MCL injuries in an animal model [10]. A protocol described by Reider et al., which included the use of a lateral hinged knee brace, weight-bearing as tolerated with crutches, immediate range of motion exercises, and progressive strength training, produced excellent results [38]. They reported a rapid return to sports with all 33 patients returning to full participation in their pre-injury sports and an early re-injury rate of 3% [38]. It is important to note that a key determinant for successful nonoperative treatment of complete MCL tears is the absence of any additional concomitant injuries including but not limited to the ACL, menisci, PMC, and POL [2,36,37,39].

Operative Management

The operative management of isolated MCL injuries is dependent on multiple factors, including location, severity, and associated injuries [40-45]. The timing of the injury is also important, as the indications for surgical treatment are different for acute and chronic injuries [40]. In the acute setting, surgery is often reserved for specific circumstances such as multi-ligament knee injuries, bony avulsions, intra-articular ligamentous entrapment, the presence of AMRI on physical examination, or complete tibial-sided avulsions in high-level athletes [10,40]. Consideration should also be given to the operative treatment of acute medial knee injuries that present with gapping in extension as surgery has been associated with improved outcomes [2]. In addition, a tibial-sided tear of the MCL can displace outside the pes anserine tendons and is unable to heal back to its anatomic insertion. Analogous to an ulnar collateral ligament injury in the thumb, a “Stener lesion of the knee” necessitates operative treatment to heal appropriately [41].

Subacute and chronic injuries may warrant surgical management if persistent pain and instability (including rotatory and/or side-to-side instability) exist despite adequate nonoperative treatment. Full-length weight-bearing radiographs should be obtained for patients with chronic medial-sided knee injuries [42]. Patients with bony valgus alignment may require a varus-producing distal femoral osteotomy before ligament reconstruction to correct their lower extremity mechanical axis and avoid a stance phase valgus wobble phenomenon [42]. If the valgus malalignment is not corrected, the reconstruction graft has a high risk of stretching out, which may result in recurrent instability and operative failure [10,42].

Surgical options can be broadly grouped into direct repair, primary repair with augmentation, advancement, or reconstruction with allograft or autograft tendon [8,16,43-45]. With chronic injuries of the sMCL, dMCL, POL, or PMC, direct repair of medial knee structures is typically not possible or recommended due to the poor tissue quality, and therefore surgical reconstruction is recommended. Internal bracing through the use of braided suture tape and knotless bone anchors has been described as a technique to reinforce the reconstruction [46]. This concept has seen recent success in the shoulder, hand, and ankle and serves to protect a repaired ligament during early rehabilitation [45]. A recent study found repair of the MCL and POL with internal bracing to be biomechanically similar to allograft reconstruction; however, long-term clinical outcomes are lacking [46].

**Figure 2:**
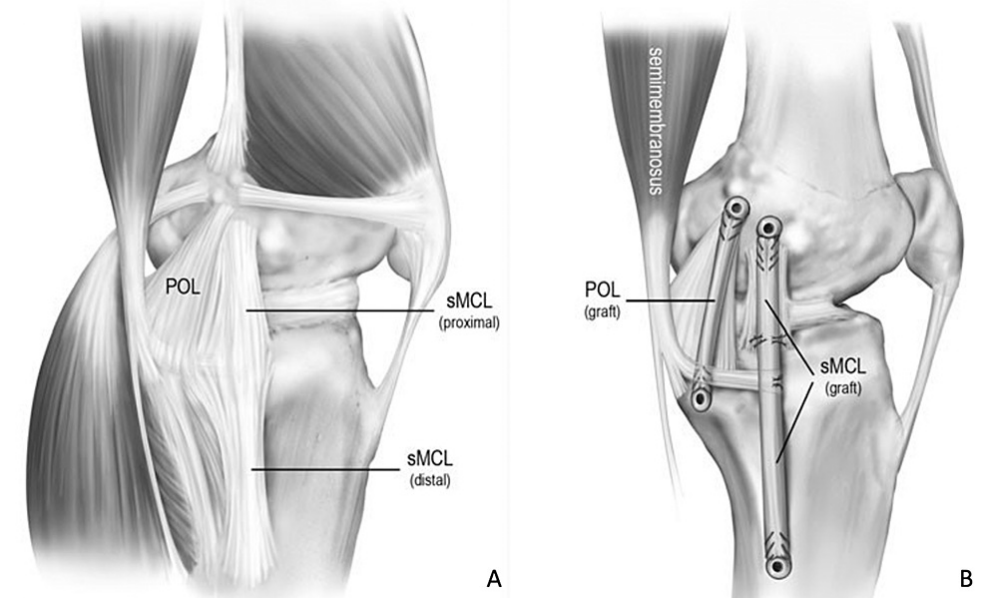
Illustration of a left knee depicting the medial knee native anatomy (Panel A) and reconstruction technique (Panel B). The sMCL and POL are reconstructed using two separate grafts and four reconstruction tunnels. The proximal tibial attachment of the sMCL is recreated by suturing the sMCL graft to the anterior arm of the semimembranosus muscle. sMCL = superficial medial collateral ligament; POL = posterior oblique ligament. Adapted with permissions from Coobs BR, Wijdicks CA, Armitage BM, Spiridonov SI, Westerhaus BD, Johansen S, Engebretsen L, LaPrade RF. An in vitro analysis of an anatomical medial knee reconstruction. Am J Sports Med. 2010;38:339-347 [16].

Numerous reconstruction techniques have been used, including combined repair and reconstruction, sMCL reconstruction, and combined MCL and POL reconstruction [16,43,47,48]. Our preferred technique when surgical intervention is warranted in acute or chronic medial knee injuries is an anatomic reconstruction of both divisions of the sMCL and the POL (Figure 2).

This technique, which has been biomechanically validated to restore native stability, utilizes two separate grafts and four graft tunnels [16]. One graft is fixed into a femoral-based tunnel at the attachment site of the sMCL and then anchored into a tibial-based tunnel at the attachment site of the distal division of the sMCL, approximately 6 cm distal to the joint line [16]. A suture anchor is then placed at the proximal sMCL attachment site and the graft is sutured to the soft tissues, recreating the proximal division of the sMCL. A second graft is fixed to the femur and tibia similarly at the anatomic attachment sites of the POL [16]. Based on the results of previous biomechanical studies, the sMCL is tightened at 20° of knee flexion and the POL is tightened at 0° of flexion [12,13,49].

The outcomes of surgical treatment, including both repair and reconstruction, have been favorable overall [43,47-51]. Hughston et al. reported excellent short-term and long-term results following acute surgical repair for AMRI, with 94% of patients returning to their pre-injury level of athletic performance [50]. In a study comparing repair versus reconstruction of the MCL and POL, Stannard et al. reported a 20% (5/25) failure rate among the repair group versus a 4% (2/48) failure rate for the reconstruction group [51]. LaPrade et al. noted excellent results in 28 patients following anatomic reconstruction of the sMCL and POL using the technique discussed above. The mean subjective International Knee Documentation Committee (IKDC) scores improved from 44 to 76 postoperatively, and all patients noted resolution of side-to-side instability symptoms. Valgus stress radiographs improved from 6.2 mm to 1.3 mm of medial compartment gapping compared with the contralateral normal knee [49]. As a result of the vast heterogeneity present among published studies, comparisons between reported results, along with definitive recommendations, are difficult to make. This plight was evident in a recent systematic review of reconstruction techniques for MCL and PMC injuries, which analyzed 25 separate studies describing 28 different techniques. Due to the lack of standardized literature, the authors of the systematic review concluded that no single technique has demonstrated clinical superiority; however, the anatomic double-bundle reconstruction of the MCL and POL resulted in less medial joint space gapping as compared to other techniques [52].

Rehabilitation

Historically, postoperative management after medial repair or reconstruction consisted of prolonged immobilization in a cast [1-3]. However, similar to current nonoperative management, many postoperative rehabilitation protocols, including the one utilized at our institution, involve early knee range of motion to prevent intra-articular adhesions and quadriceps atrophy [49-51]. The patient is placed in a hinged knee brace and is made non-weight bearing for the first six weeks [49-51]. The surgeon needs to determine intra-operatively the “safe zone,” which is the motion arc that does not place undue stress on the repair or reconstruction, and communicate this to the physical therapist [50,51]. Range of motion within the “safe zone” can be started on postoperative day one and should not be exceeded for the first two weeks. Simple strengthening exercises are encouraged immediately after surgery. After two weeks, the range of motion is increased as tolerated; however, the internal and external tibial rotation should be avoided for the first few months [1].

Weight-bearing is allowed after six weeks, at which time closed-chain kinetic exercises are permitted for functional strengthening with the addition of two-limb support squatting limited to 70° of knee flexion [50,51]. Restoration of normal gait mechanics is emphasized and the patient is educated about avoiding pivoting motions on a planted foot, which could stretch out the grafts. Basic agility and plyometric exercises can be initiated 16 to 20 weeks after surgery. Following completion of the rehabilitation program, return to sports may be permitted provided the patient can pass sport-specific functional tests and objective evidence of knee stability is present on clinical examination [50,51].

## Conclusions

The sMCL is the most commonly injured structure in the knee. A detailed history and clinical examination, along with appropriate imaging, are imperative in the evaluation of these injuries. Consideration of the role of the dMCL, POL, and medial meniscus can help avoid missing associated injuries. The majority of acute MCL injuries can be successfully managed nonoperatively with good outcomes. Failure to diagnose and treat concomitant injury to the posteromedial corner can result in residual instability of the knee. Early range of motion, functional bracing, and rehabilitation are the cornerstone of treatment for most grade I and II injuries. Treatment of isolated grade III injuries is more complex with less predictable outcomes for nonoperative treatment. When surgical management is warranted, multiple different repair and reconstruction techniques have been used with successful results. The recently developed anatomic reconstruction technique may restore the native stability and load-sharing relationships among the medial knee structures and should be considered for operative cases. Further high-quality studies are required to determine which technique provides superior results.
